# Successful Re-ileostomy Using Skin Flap Formation Techniques

**DOI:** 10.7759/cureus.74940

**Published:** 2024-12-01

**Authors:** Hitomi Matsuki, Shoji Oura, Naoki Kataoka

**Affiliations:** 1 Department of Surgery, Kishiwada Tokushukai Hospital, Kishiwada, JPN

**Keywords:** ileostoma, low height stoma, re-ileostomy, skin complication, skin flap formation technique, stoma re-making

## Abstract

A 61-year-old woman underwent an emergent operation with sigmoid colon cancer resection, colostomy, and ileostomy on colon perforation. The low ileostoma, caused by intra-abdominal bad conditions, had irritated the surrounding skin after surgery, intermittently forcing the patient to fast for a certain period. Six months after the operation, under the judgment that re-ileostomy, essential for hospital discharge, seemed very difficult through another laparotomy, we attempted to make the ileostoma higher not with pulling the ileum from the abdomen but with lowering the surrounding skin using skin flap formation techniques. For re-ileostomy, we cored out the ileostoma to the external oblique muscle, followed by wide skin flap formation. Then, we lowered the peri-stomal skin level with subcutaneous fat resection. Finally, we sutured the skin flap to the ileostoma base. The skin defect area on the side wall of the ileostoma caused surgical site infection but shrank over time, finally leading to the fusion between the ileal mucosa and the ileostoma base skin. The patient has been well without major events, eating a normal diet for eight months after the re-ileostomy. In conclusion, general surgeons should note that this type of stoma re-making is a feasible and minimally invasive alternative to conventional stoma re-making through another laparotomy.

## Introduction

Ileostomas are created for various pathological conditions such as colorectal obstruction or possible suturing failure at the anastomotic site after colorectal cancer surgery [1]. Compared to colostomas, ileostomas are more likely to be created temporarily but are not uncommon to be created permanently.

Feces from an ileostoma are much more watery than those from a colostoma [2-4]. It, therefore, is necessary to pay more attention to skin complications after ileostomy than those after colostomy [5]. Furthermore, low ileostomy due to technical problems or abdominal conditions is associated with more stoma-related complications. Especially, skin complications around an ileostoma sometimes force general surgeons to re-make it. Re-ileostomy, however, is generally more difficult than the primary ileostomy and is sometimes highly invasive to the patients.

We herein report a case of successful re-ileostomy with a minimally invasive surgical method using skin flap formation techniques [6].

## Case presentation

**Figure 1:**
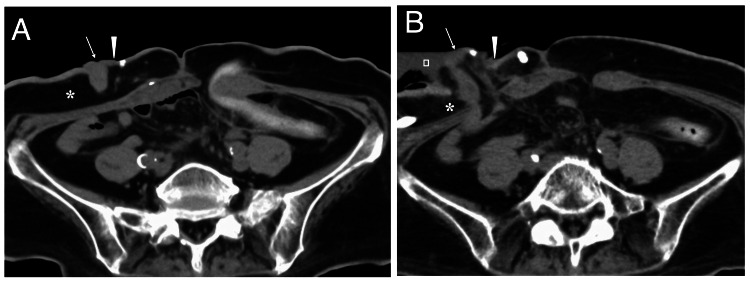
Computed tomography (CT) of and around the ileostoma A: CT showed that subcutaneous fat was thick as 3 cm (asterisk), and the top of the ileostoma (arrow) was located at the same level of the ileostoma base (arrowhead) and the surrounding skin before reoperation; B: although watery feces (open square) partially obscured the ileostoma contour, CT showed the ileostoma (arrow) was much higher than the ileostoma base (arrowhead). Subcutaneous fat around the ileostoma (asterisk) was thinner than that before reoperation.

A 61-year-old woman with abdominal pain was referred to our hospital. Computed tomography (CT) showed intra-abdominal free air and a presumed sigmoid colon mass, highly suggesting the colon perforation due to sigmoid colon cancer. Diagnostic laparotomy showed a hard and large sigmoid colon mass, markedly dilated descending colon, and strong invasion or adhesion between the sigmoid colon mass and the surrounding organs such as the ascending colon, ileum, and retroperitoneum. Under the tentative diagnosis of colon cancer-induced perforation, we did emergent surgery on the patient and found the sigmoid colon forming a large mass involving the surrounding organs hardly to be dissected. We, therefore, resected the presumed sigmoid colon cancer and the involved surrounding organs without lymph node dissection, followed by stoma creation both at the sigmoid colon and the ileum. Although the colostoma was created without any problems, less extendability of the mesoileum unfortunately resulted in the limited-height ileostoma creation. The top of the ileostoma, therefore, was unfortunately located at the same level as the neighboring abdominal skin (Figure 1A).

**Figure 2:**
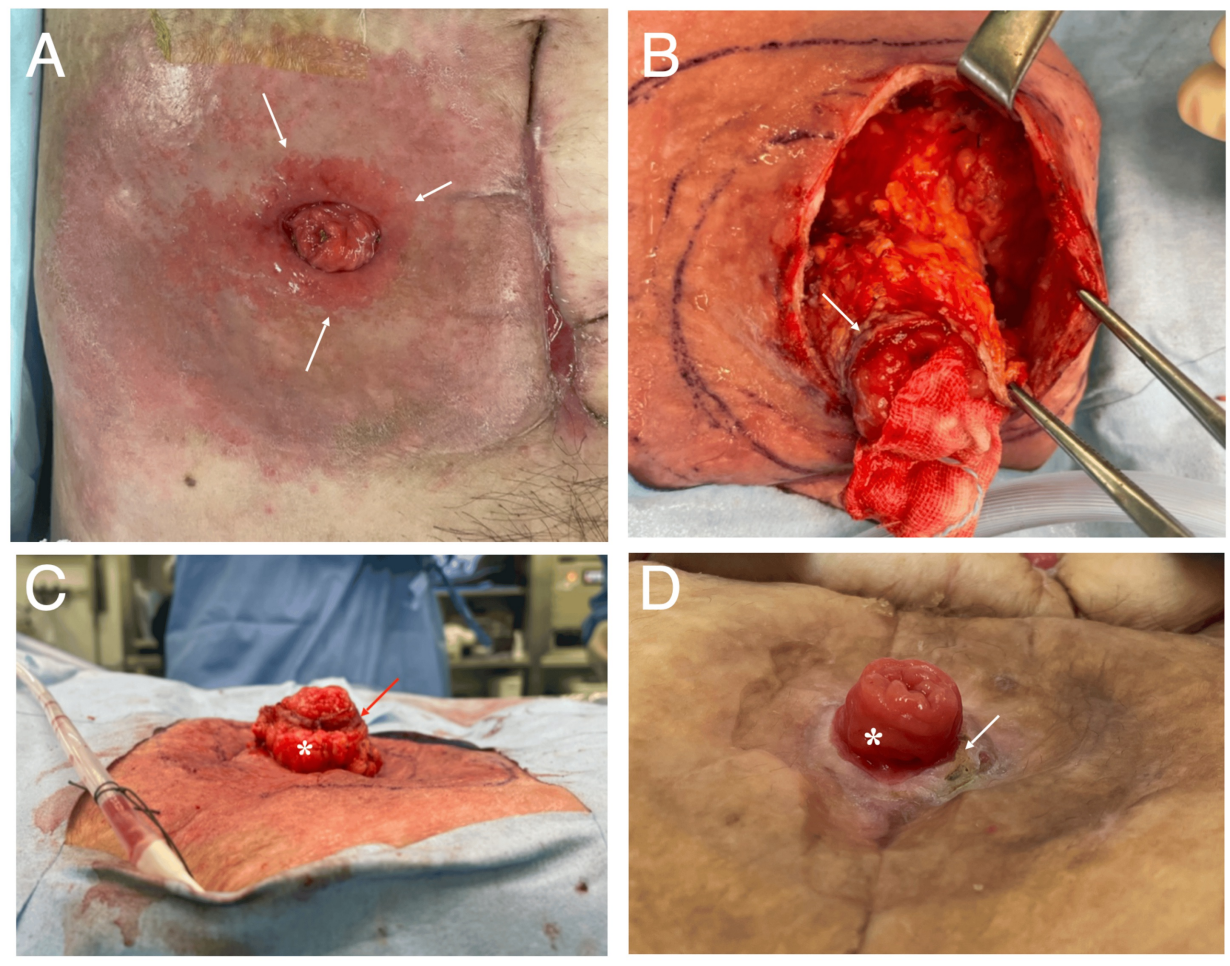
Ileostoma status and intra-operative findings A: the low height ileostoma caused painful inflammation on the surrounding skin (arrows); B: skin flap was radially made, approximately 8 cm in size (the arrow indicated the minimal skin remnant attached to the ileostoma); C: a lateral view clearly showed that the peri-stomal skin remnant (arrow) was located at a much higher level than the re-ileostomy base skin; D: as the skin defect area had gradually shrank on wound healing process, the ileal mucosa (asterisk) had been gradually drawn up from the abdomen, and the remnant  peri-stomal skin was fused with the skin flap proximal edges (arrow). Ileostoma showed a very good condition six months after the reoperation.

This low-height ileostoma postoperatively caused prolonged skin complications, i.e., painful skin redness (Figure 2A) and often intermittently forced the patient to fast for a certain period.

**Figure 3:**
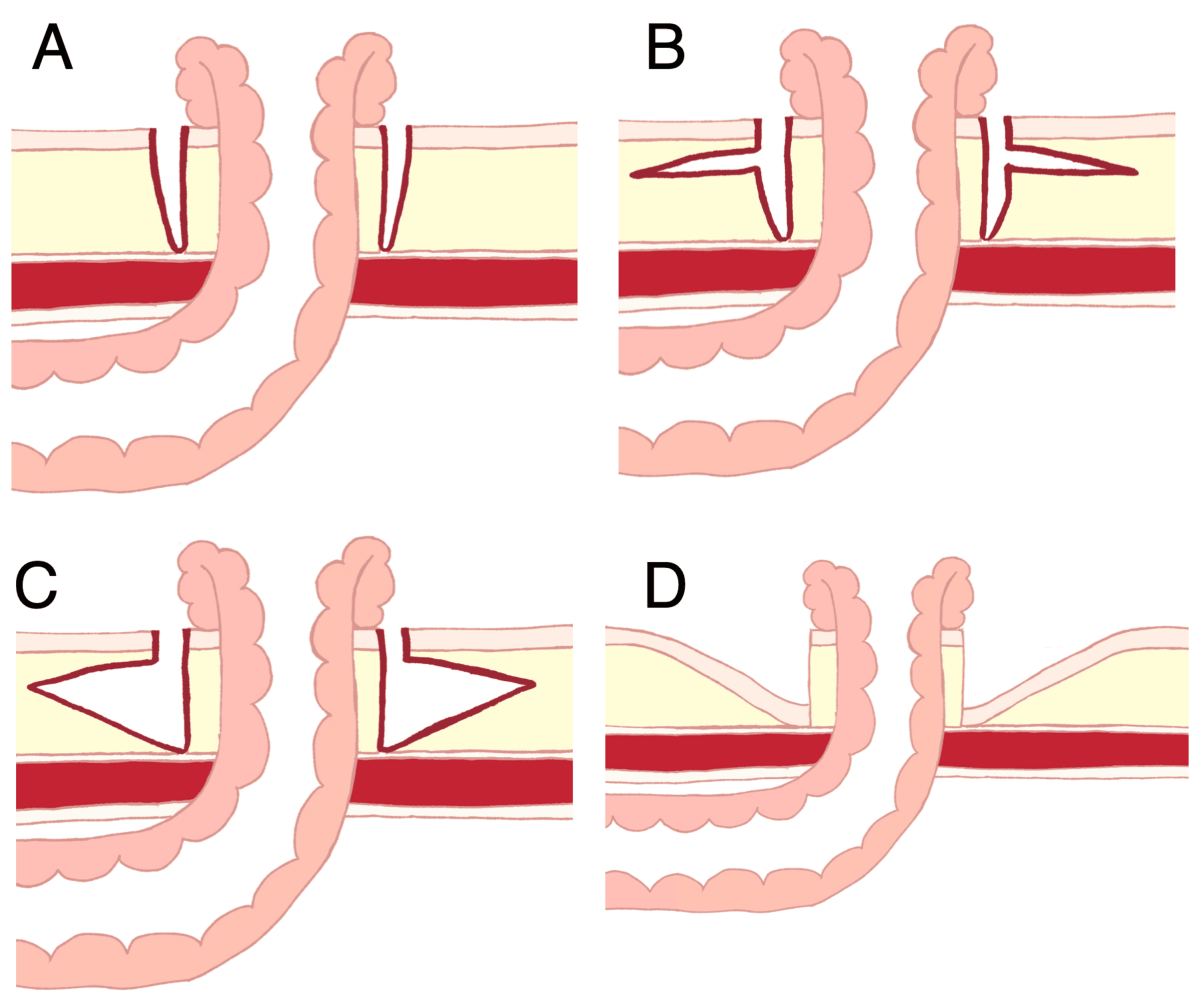
Illustrations of operative procedures A: ileostoma, both with the surrounding minimal skin and fat, was cored out to the external oblique muscle; B: a skin flap was made approximately 8 cm in size; C: peri-stomal abdominal fat was resected circumferentially in a triangle shape from the ileostoma base to the distal edges of the skin flap; D: the proximal edges of the skin flap were circumferentially sutured to the ileostoma base after the resection of redundant skin. # These images were illustrated by Ms. Amane Nakamura, Kishiwada Tokushukai Hospital, for this paper.

Improvement of the ileostomy condition was judged to be essential for the patient discharge. It, however, appeared to be highly challenging through a subsequent laparotomy. We, therefore, came up with the idea of re-ileostomy with skin lowering and attempted it six months after the operation. For re-ileostomy, we firstly cored out the ileostoma, both with the surrounding minimal skin and fat to the external oblique muscle (Figure 3A), followed by wide skin flap formation up to the expected pouch attachment edges (Figures 2B, 3B). Then, we lowered the peri-stomal skin level with circumferential subcutaneous fat resection in a triangle shape from the ileostoma base to the distal skin flap edges (Figure 3C). Finally, we sutured the proximal skin flap edges to the ileostoma base after the redundant skin resection (Figures 2C, 3D).

All these procedures led to a successful re-ileostomy (Figures 1B, 2C) but left a large skin defect on the side of the ileostoma (Figure 2C). The patient recovered from the skin complications shortly after the re-ileostomy and resumed a normal diet a week after the reoperation. In addition, the patient has not developed any skin complications, except for surgical site infection due to the re-ileostomy-induced skin defect, since then. On the wound healing process, the large skin defect gradually shrank and finally resulted in the complete fusion of the original peri-stomal skin and the skin flap proximal edges, leading to the disappearance of the skin defect in three months (Figure 2D). The patient has been well without cancer- or stoma-related major events and has been eating a normal diet for eight months after the re-ileostomy.

## Discussion

Prolonged skin complications [7] observed in this case were mainly caused by the very low height ileostoma, which was caused not by technical failure but by bad conditions of the intra-abdominal organs. We, therefore, were highly concerned that re-ileostomy through another open/laparoscopic surgery could cause severe visceral damage during the operation. To overcome the potential complications, we came up with an idea that the ileostoma could be re-made not by pulling the ileum from the abdomen, but by lowering the peri-stomal skin level. We, therefore, made a wide skin flap, commonly done in breast cancer surgeries, and resected the subcutaneous fat to lower the peri-stomal skin level. Reoperation caused an unfortunate surgical site infection temporally but gave the ileostoma sufficient height and promptly eliminated the skin complications.

In breast cancer surgery, much attention should be paid to subcutaneous fat preservation in a thick flap manner for better local control [8]. However, skin flap formation techniques on re-ileostomy need no attention on how to preserve the subcutaneous fat. These surgical techniques, therefore, seem very easy even for surgical trainees and can provide a feasible alternative to re-ileostomy especially for patients with thick peri-stomal subcutaneous fat and possible severe adhesions under the stoma.

Epithelia such as skin and mucosa have a role as a barrier to the bacteria and viruses [9]. In fact, the skin defect after the re-ileostomy caused surgical site infection and delayed the patient discharge. On the other hand, the skin defect had naturally shrunk on wound healing, leading to the drawing up of the ileal mucosa. Although the ileum could not even be fully pulled up on the initial surgery, it is noteworthy that the mucosa was gradually but fully drawn up during the healing process of the skin defect of the ileostoma.

## Conclusions

On abdominal surgery, especially emergency surgery, general surgeons often have the opportunity to add a covering stoma. However, depending on the condition of the abdominal organs, attending surgeons are forced to make poor ileostomas, sometimes requiring re-ileostomy due to ileostoma-induced complications. In such cases, it is often difficult to re-create it through another open/laparoscopic surgery. General surgeons should note that stoma re-making using skin flap formation techniques is a feasible and minimally invasive alternative to conventional stoma re-making.

## References

[1] Solitano V, Vuyyuru SK, Yuan Y (2024). Management of complications in patients with an ileostomy: an umbrella review of systematic reviews for the EndOTrial Consortium. Int J Colorectal Dis.

[2] Hendren S, Hammond K, Glasgow SC, Perry WB, Buie WD, Steele SR, Rafferty J (2015). Clinical practice guidelines for ostomy surgery. Dis Colon Rectum.

[3] Messaris E, Sehgal R, Deiling S, Koltun WA, Stewart D, McKenna K, Poritz LS (2012). Dehydration is the most common indication for readmission after diverting ileostomy creation. Dis Colon Rectum.

[4] Hayden DM, Pinzon MC, Francescatti AB (2013). Hospital readmission for fluid and electrolyte abnormalities following ileostomy construction: preventable or unpredictable?. J Gastrointest Surg.

[5] Shabbir J, Britton DC (2010). Stoma complications: a literature overview. Colorectal Dis.

[6] Haagensen CD, Bodian C (1984). A personal experience with Halsted's radical mastectomy. Ann Surg.

[7] Persson E, Berndtsson I, Carlsson E, Hallén AM, Lindholm E (2010). Stoma-related complications and stoma size - a 2-year follow up. Colorectal Dis.

[8] Li W, Liang Y (2024). Retrospective Analysis: Preserving More Subcutaneous Tissue Shows no Increase the Risk of Breast Cancer Recurrence. Technol Cancer Res Treat.

[9] Wertz PW (2023). Synopsis of barrier function of skin and mucosa—Volume 2. Int J Mol Sci.

